# Supplementary material for: Content-rich biological network constructed by mining PubMed abstracts
Source: BMC Bioinformatics. 2004 Oct 8;5:147. doi: 10.1186/1471-2105-5-147 (PMC528731; doi:10.1186/1471-2105-5-147)
Supplement: Additional File 2 — The original results of the above study (non-essential files are deleted to keep the file size under the limit set by BMC bioinformatics). [file 1471-2105-5-147-S2.bz2 › chilibotAdditionalFile2/dip50/332ID8387891E3928/html/TNFRSF1A_LTA.html]

 


 **TNFRSF1A** and **LTA** 
  
Found 102 abstracts in PubMed, retrieved 50.  
 

 What does Google say? 
 PDF only 
| .edu only 

---

**Interactive relationship** (e.g. stimulation, inhibition, etc)

**Neutral relationship**- Since this was probably due to the complex immunomodulatory effects of TNF and lymphotoxin alpha  [ **LTA** ]  signaled via TNFR1  [ **TNFRSF1A** ]  on lymphohemopoietic cells, adoptive transfer experiments with spleen cells from diabetic NOD mice were conducted.  Ref: 10201999 J Immunol, 1999
- TNF and LT alpha  [ **LTA** ]  both bind to the tumor necrosis factor receptors TNFR  [ **TNFRSF1A** ] .  Ref: 12163535 J Neurophysiol, 2002
- LT consists of soluble LT alpha  [ **LTA** ]  3, which binds to TNF R1  [ **TNFRSF1A** ]  and TNF R2, and membrane LT alpha  [ **LTA** ]  1 beta 2, which binds to LT beta R.  Ref: 12882833 Int Immunol, 2003
- As the TNF beta  [ **LTA** ]  TNFR1  [ **TNFRSF1A** ]  co crystal structure was the first in the family to be solved, rational drug design based on the ligand receptor interaction sites is more advanced.  Ref: 11467075 Curr Drug Targets, 2000
- The action of LT alpha  [ **LTA** ]  in lymphoid organogenesis is mediated mostly by the membrane form of LT by a mechanism independent of TNF receptor I TNFR  [ **TNFRSF1A** ]  I or II TNFR  [ **TNFRSF1A** ]  II.  Ref: 10687308 J Med Invest, 1999
- TNF alpha and LT alpha  [ **LTA** ]  are both members of the TNF family of molecules and cause cell death by apoptosis following interaction with their counter receptors, the TNFR1  [ **TNFRSF1A** ]  and TNFR2, leading to a cascade of proteolytic events culminating in the blebbing of the cytoplasmic membrane, nuclear condensation and DNA fragmentation.  Ref: 11140465 Arch Immunol Ther Exp Warsz , 2000
- Four members of the tumor necrosis factor TNF ligand family, TNF alpha, LT alpha  [ **LTA** ] , LT beta, and LIGHT, interact with four receptors of the TNF nerve growth factor family, the p55 TNF receptor CD120a  [ **TNFRSF1A** ] , the p75 TNF receptor CD120b, the lymphotoxin beta receptor LT beta R, and herpes virus entry mediator HVEM to control a wide range of innate and adaptive immune response functions.  Ref: 10358762 Annu Rev Immunol, 1999

**Non-interactive relationship** (e.g. studied together, co-existance, homology, etc.)

- Our results indicate that LT alpha  [ **LTA** ]  induced inflammation is dependent on the interaction of LT alpha3 with TNFR1  [ **TNFRSF1A** ] , and there is no obvious role for TNFR2, since in its absence, LT alpha  [ **LTA** ]  induced inflammation is quantitatively and qualitatively similar to that seen in the wild type.  Ref: 9552007 J Immunol, 1998
- We have previously demonstrated, using expressed deletion constructs, that the fourth membrane proximal cysteine rich repeat of the p55 TNF receptor TNF R  [ **TNFRSF1A** ]  is not required for binding of tumour necrosis factor alpha TNF or lymphotoxin alpha  [ **LTA** ]  LT.  Ref: 9831174 Eur Cytokine Netw, 1998
- Immunity to Toxoplasma gondii critically depends on TNFR  [ **TNFRSF1A** ]  type I mediated immune reactions, but the precise role of the individual ligands of TNFR1  [ **TNFRSF1A** ] , TNF and lymphotoxin alpha  [ **LTA** ]  LTalpha, is still unknown.  Ref: 12794148 J Immunol< MedlineTA>J Immunol, 1998
- METHODS Immunohistochemical IHC staining was performed on formalin fixed, paraffin embedded tissues from 39 bronchogenic adenocarcinomas and 32 squamous cell carcinomas using polyclonal antibodies against TNF alpha, TNF beta  [ **LTA** ] , TNF R1  [ **TNFRSF1A** ] , and TNF R2 proteins.  Ref: 9669810 Cancer, 1998
- To address the question of whether the cell death inducing cytokines TNF and lymphotoxin alpha  [ **LTA** ]  are involved in this process, we generated nonobese diabetic NOD mice that are deficient for TNF receptor 1 TNFR1  [ **TNFRSF1A** ]  or TNFRp55.  Ref: 10201999 J Immunol, 1999
- The results from TNFR1  [ **TNFRSF1A** ]  IgG transgenic mice suggest that the hypermetabolic and anorectic responses induced by LPS are independently regulated since, in the absence of TNF alpha or LT alpha  [ **LTA** ] , the LPS induced hypermetabolism is almost prevented but not the anorexia.  Ref: 11125311 Eur Cytokine Netw, 2000
- In the present study, the expression of TNF, LT alpha  [ **LTA** ]  the soluble form of LT, LT beta the beta chain of LT alpha  [ **LTA** ]  beta, the membrane bound form of LT, TNF RI  [ **TNFRSF1A** ] , TNF RII, LT beta R, FasL, and Fas receptor in MS lesions has been examined by immunohistochemistry for protein and by RT PCR for mRNA.  Ref: 9809372 Rev Neurol Paris , 1998
- To explore the latter possibility, the course of disease was examined in infected mice lacking TNFR  [ **TNFRSF1A** ]  I and or TNFRII, TNF only, or both TNF and lymphotoxin alpha  [ **LTA** ] .  Ref: 10415054 J Immunol, 1999
- TNFR  [ **TNFRSF1A** ]  I deficient BM was able to restore FDC organization and GC formation in LT alpha  [ **LTA** ]  deficient mice, indicating that formation of these structures does not require TNFR  [ **TNFRSF1A** ]  I expression on BM derived cells.  Ref: 9396768 J Exp Med, 1997
- These data show a crucial role for the secreted LT alpha3 signaling via TNFR1  [ **TNFRSF1A** ]  in LT alpha  [ **LTA** ]  induced inflammation, and a separate and distinct role for the membrane LT alpha1beta2 form in this inflammatory process.  Ref: 9552007 J Immunol, 1998
- To study the role of tumour necrosis factor alpha TNF alpha on some of these metabolic responses to endotoxin, we have used transgenic mice expressing soluble tumour necrosis factor receptor 1 IgG fusion protein TNFR1  [ **TNFRSF1A** ]  IgG as well as TNF alpha knockout KO, lymphotoxin alpha  [ **LTA** ]  LT alpha  [ **LTA** ]  KO, and interferon gamma receptor IFN gamma R KO mice.  Ref: 11125311 Eur Cytokine Netw, 2000
- This report analyzes the gene expression of bcl xs, bcl xl, bax alpha, bax beta, fadd, fas, fas ligand fas L, ice, TNF alpha, TNF beta  [ **LTA** ] , TNFR1  [ **TNFRSF1A** ] , TNFR2, TRAF1, TRAF2, TRAF3, cIAP2, and tradd at the level of mRNA in the single Reed Sternberg cells and their variants.  Ref: 9516144 Blood, 1998
- In mice deficient in either lymphotoxin alpha  [ **LTA** ]  LT alpha  [ **LTA** ]  or type I tumor necrosis factor receptor TNFR  [ **TNFRSF1A** ]  I, organized clusters of follicular dendritic cells FDC and germinal centers GC are absent from the spleen.  Ref: 9396768 J Exp Med, 1997
- Results demonstrated that rejection of the B16.MUC1 tumor cell line was primarily mediated by CD4 T cells, and required Fas ligand, lymphotoxin alpha  [ **LTA** ] , CD40, CD40 ligand and CD28, but not perforin, gammadelta T cells, IL 4, IL 10, IL 12 or tumor necrosis factor receptor 1  [ **TNFRSF1A** ] .  Ref: 12147624 Int Immunol, 2002
- The serum concentrations of tumor necrosis factor beta  [ **LTA** ]  TNF beta  [ **LTA** ] , TNF receptor 1 TNFR 1  [ **TNFRSF1A** ] .  Ref: 11939943 Acta Neurol Scand, 2002
- RESULTS Significant coexpression of TNF alpha, TNF beta  [ **LTA** ] , TNF R1  [ **TNFRSF1A** ] , and TNF R2 was observed in NSCLC significance range, P <.  Ref: 9669810 Cancer, 1998
- The data in this study demonstrate that FDC organization and GC formation are controlled by both LT alpha  [ **LTA** ]  expressing BM derived cells and by TNFR  [ **TNFRSF1A** ]  I expressing non BM derived cells.  Ref: 9396768 J Exp Med, 1997
- The different expression patterns of the two distinct TNFR  [ **TNFRSF1A** ]  homologues may be critical in determining whether binding with TNF alpha or TNF beta  [ **LTA** ]  have activating, proliferative, or apoptotic effects on target cells.  Ref: 12631519 Dev Comp Immunol, 2003
- We investigated the role of LT alpha  [ **LTA** ]  and TNFR  [ **TNFRSF1A** ]  I in the establishment of spleen FDC and GC structure by using reciprocal bone marrow BM transfer.  Ref: 9396768 J Exp Med, 1997
- In contrast to lymphotoxin alpha  [ **LTA** ]  deficient mice LTalpha, bone marrow or fetal liver transplantation did not correct the abnormal organization of the spleen, location of B cells, the lack of an FDC network, nor the antibody response in TNFR1  [ **TNFRSF1A** ]  mice.  Ref: 9463397 J Exp Med, 1998
- This activity of LT depends on membrane LT alpha  [ **LTA** ]  1 beta 2 and not soluble LT alpha  [ **LTA** ]  3, since DA virus persisted and induced inflammatory demyelination in LT beta R, but not TNF R1  [ **TNFRSF1A** ]  or TNF R2, mice.  Ref: 12882833 Int Immunol, 2003
- In this review we summarize the essential findings about the function of tumour necrosis factor TNF and its cognate receptors TNFR1  [ **TNFRSF1A** ]  and TNFR2, and lymphotoxin alpha  [ **LTA** ]  LT alpha  [ **LTA** ]  ligands in immune mediated CNS inflammation and demyelination.  Ref: 11004118 Brain, 2000
- CONCLUSIONS Although NSCLC exhibits strong coexpression of TNF alpha, TNF beta  [ **LTA** ] , TNF R1  [ **TNFRSF1A** ] , and TNF R2, there is a loss down regulation of TNF receptors in high stage tumors.  Ref: 9669810 Cancer, 1998
- Use of gene knockout mice indicates that depletion of BM B cells is dependent on TNF alpha, lymphotoxin alpha  [ **LTA** ] , and both TNF receptors, TNFR1  [ **TNFRSF1A** ]  p55 and TNFR2 p75.  Ref: 12444124 J Immunol, 2002
- The crystal structures of TNF alpha, TNF beta  [ **LTA** ] , the extracellular domain of TNFR 1  [ **TNFRSF1A** ]  denoted sTNFR 1, and the TNF beta  [ **LTA** ]  sTNFR 1 complex have been defined by crystallography.  Ref: 10891884 Microsc Res Tech, 2000
- The effect of SEB to induce EAE in IL 6 mice depends on TNF receptor type 1 TNFR1  [ **TNFRSF1A** ]  signaling because IL 6 TNF LT alpha  [ **LTA** ]  and IL 6 TNFR1  [ **TNFRSF1A** ]  are refractory to SEB.  Ref: 11477542 Eur J Immunol, 2001
- There were significant differences between HAM patients and normal controls in the distributions of TNF promoter region polymophism at position 857, the LT alpha  [ **LTA** ]  gene NcoI polymorphism, and the T G substitution in exon 6 of the TNFR  [ **TNFRSF1A** ]  2 gene.  Ref: 11163081 Hum Immunol, 2000
- The heterotrimeric lymphotoxin alpha  [ **LTA** ]  1 beta 2 LTalpha 1 beta 2 complex and LIGHT, a new member of the tumor necrosis factor TNF superfamily, have been identified as membrane anchored ligands for the LTbeta receptor LTbetaR, a member of the TNF receptor TNFR  [ **TNFRSF1A** ]  superfamily.  Ref: 11429164 J Interferon Cytokine Res, 2001
- In contrast, although Th1 cytokine receptors tumor necrosis factor TNF RI  [ **TNFRSF1A** ] , interferon IFN gammaRalpha, IFN gammaRbeta, were detected, their cytokines IFN gamma, TNF alpha, lymphotoxin alpha  [ **LTA** ]  were not.  Ref: 11810184 Acta Neuropathol Berl , 2002
- IL 2 induced tumor necrosis factor TNF beta  [ **LTA** ]  expression further analysis in the IL 2 knockout model, and comparison with TNF alpha, lymphotoxin beta, TNFR1  [ **TNFRSF1A** ]  and TNFR2 modulation.  Ref: 11157847 Int Immunol, 2001
- We studied tumor necrosis factor TNF, lymphotoxin alpha  [ **LTA** ]  LT alpha  [ **LTA** ] , and TNF receptors type 1 TNFR 1  [ **TNFRSF1A** ]  and type 2 TNFR  [ **TNFRSF1A** ]  2 gene polymorphisms as well as HLA class II DRB1 alleles in Japanese patients with human T cell lymphotropic virus type I HTLV I associated myelopathy HAM n = 51, patients with adult T cell leukemia lymphoma ATL n = 48, asymptomatic HTLV I carriers n = 50, and HTLV I seronegative, normal controls n = 112.  Ref: 11163081 Hum Immunol, 2000
- Surprisingly, both perforin and FasL played unique roles during the effector phase of immunity to Panc02.MUC1, while lymphotoxin alpha  [ **LTA** ] , but not TNFR 1  [ **TNFRSF1A** ] , was required for immunity against Panc02.MUC1 tumors.  Ref: 12111121 Cancer Immunol Immunother, 2002
- The results suggest that the TNF LT alpha  [ **LTA** ]  gene region within the HLA class III of chromosome 6 and the TNFR  [ **TNFRSF1A** ]  2 gene region located on chromosome 1p36 might contribute to susceptibility to HAM,.  Ref: 11163081 Hum Immunol, 2000
